# Paternalistic persuasion: are doctors paternalistic when persuading patients, and how does persuasion differ from convincing and recommending?

**DOI:** 10.1007/s11019-023-10142-2

**Published:** 2023-03-01

**Authors:** Anniken Fleisje

**Affiliations:** Centre for the Study of Professions, Oslo Metropolitan University, Oslo, Norway

**Keywords:** Medical paternalism, Persuasion, Overriding treatment preferences, Doctor-patient communication

## Abstract

In contemporary paternalism literature, persuasion is commonly not considered paternalistic. Moreover, paternalism is typically understood to be problematic either because it is seen as coercive, or because of the insult of the paternalist considering herself superior. In this paper, I argue that doctors who persuade patients act paternalistically. Specifically, I argue that trying to *persuade* a patient (here understood as aiming for the patient to consent to a certain treatment, although he prefers not to) should be differentiated from trying to *convince* him (here understood as aiming for the patient to *want* the treatment) and recommending (the doctor merely providing her professional opinion). These three forms of influence are illustrated by summaries of video-recorded hospital encounters. While convincing and recommending are generally not paternalistic, I argue that persuasion is what I call *communicative paternalism* and that it is problematic for two reasons. First, the patient’s preferences are dismissed as unimportant. Second, the patient might wind up undergoing treatment against his preferences. This does not mean that persuasion always should be avoided, but it should not be undertaken lightly, and doctors should be aware of the fine line between non-paternalism and paternalism. The fact that my analysis of paternalism differs from traditional accounts does not imply that I deem these to be wrong, but rather that paternalism should be considered as a more multi-faceted concept than previous accounts allow for.

## Introduction

Paternalism can, very generally, be characterized as overriding another’s preferences with the intention of benefitting them or protecting them from harm. The paternalist, thus, substitutes their judgment for that of the person interfered with. Until the second half of the twentieth century, doctors commonly behaved paternalistically by making decisions without involving their patients (Katz 1984). Although medical practices have since evolved to include greater patient involvement, paternalism in healthcare still is not uncommon. Compulsory treatment is a clear example, while lying is another.

However, what should be viewed as paternalism in doctor-patient conversations––for example, in discussions about treatment options––is less clear. If a doctor persuades a patient to consent to a treatment that the patient is reluctant to proceed with, is she^1^ acting paternalistically? And if so, is this problematic?

In contemporary philosophical literature on paternalism, a common view is that persuading another by providing reasons or arguments is not paternalistic (Tsai 2014).^2^ Persuasion, according to this view, is not paternalistic because the person doing the persuading shows respect for the other’s reasoning capacities, while paternalism is viewed as overriding the other in a way that does not respect their capacity to reason on their own (Shiffrin 2000). Most theorists consider paternalism morally problematic but agree that it is acceptable in certain contexts. The problem with paternalism is often considered to be that it undermines autonomy. In motive-based accounts, however, the paternalist’s motive is emphasized and the problem with paternalism, from this perspective, is that the paternalist is placing themselves in a superior position to judge what is best for the other (Begon 2016).

This paper focuses on patients who are reluctant about the treatment course that their doctors think best. I argue that *persuading* a patient into treatment is different from *convincing* them, which, in turn, differs from *recommending*. I will use real-life hospital encounters to illustrate and discuss the differences. While offering treatment recommendations or convincing patients to opt for certain treatments generally is not paternalistic, I argue that doctors who persuade patients are acting paternalistically. I term this form of paternalism *communicative paternalism*. In contrast to motive-based accounts of paternalism, the problem with this form of paternalism, I argue, is *not* that the doctor is placing herself in a superior position to judge what is best for the patient. Rather, the problems are, first, that the patient’s preferences are dismissed during the treatment conversation, and second, that the patient might undergo treatment that he does not want. Whereas both dismissal of preferences and unwanted treatment undermine patient autonomy, I argue that the latter is more problematic than the former. This does not mean that it is necessarily wrong to persuade patients, but that the issue of persuasion should not be taken lightly.

Below, I will discuss different forms of influence and how I believe they should be understood, focusing, specifically, on persuading, convincing, and recommending. When the differences between these three forms of influence are established, I will argue that persuasion––unlike convincing and recommending––is paternalistic. I then explain what I consider most problematic with the paternalism inherent in persuasion, followed by the paper’s conclusion.

### Forms of influence: persuading, convincing, and recommending

Faden and Beauchamp speak of persuasion as one of three main forms used to influence another person. They construct a continuum ranging from controlling to non-controlling influences (1986, 259) and place coercion at the “controlling” end of the spectrum, persuasion at the “non-controlling” end, and manipulation in the middle, as approximately depicted below:
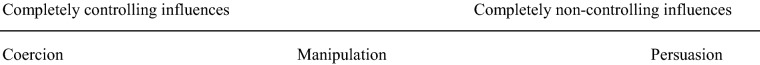


When, according to Faden and Beauchamp, someone is persuaded they come to believe something through reason––as opposed to emotion––and freely accept the persuader’s beliefs as their own. Coercion is characterized as an intentional and irresistible threat, while manipulation is used as an umbrella term for influence that is neither coercion nor persuasion (Faden and Beauchamp 1986), for example, lying, framing information, or using a particular tone of voice (Beauchamp and Childress 2019).

Oxman et al. present an adjusted version of the continuum in the context of public health communication. They place *inform* (providing the pros and cons of each treatment option)––instead of *persuade*––at what we may call the “non-controlling” end. Then comes *recommend*, which they define as to “suggest or advise a decision based on explicit reasons,” followed by *persuade*, defined as “influence by reason and argument” (Oxman et al. 2022, 3).

Adding *informing* and *recommending* brings nuance to the continuum, but I believe further nuances are needed. Faden and Beauchamp’s umbrella term for manipulation, for example, is arguably too wide as, say, deliberate lying is substantially and morally quite different from using a particular tone of voice and framing information in a certain way, which may not be deliberate (and is potentially unavoidable). The category of coercion could also be problematized. However, coercion and threats belong (hopefully!) to rare newspaper stories and philosophers’ thought experiments, and manipulation and its ethical dimensions are discussed widely elsewhere (for an overview, see, e.g., Noggle 2022). Therefore, I will focus mainly on influence in the middle and the “non-controlling” end of the continuum. Below, I will distinguish *persuading* from what I term *convincing*, as well as pointing out how the latter, in my view, differs from recommending. By clarifying the concepts of recommending, convincing, and persuading, subtle aspects of verbal influence can be identified. Moreover, it will become evident how a conversation can develop from a doctor merely providing a professional opinion, which is essentially non-paternalistic, into a case of paternalism.

Placing the abovementioned forms of influence on the continuum may look something like this:
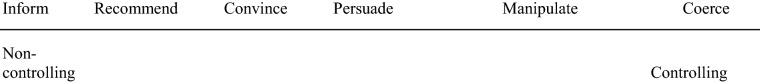


As we shall see, the continuum can also illustrate a stepwise process in time.^3^ The doctor might begin by informing about, or recommending, a certain treatment. If the patient does not immediately choose the recommended option, the doctor then might try to convince him, and then––if he remains reluctant––try to persuade him.

In the discussion below, three forms of influence––recommending, convincing, and persuading––will be illustrated by summaries of video-recorded doctor-patient encounters at a Norwegian hospital (for a full account of the sampled material, see Jensen et al. 2011). Approximately 200 recorded encounters were reviewed, 10 of which were categorized as encounters in which the patient expressed reluctance toward the doctor’s medical suggestions,^4^ and these encounters were transcribed verbatim. Three of these 10 cases are summarized below. In all three cases, the doctors provide reasons in favour of the treatment courses that they considered to be the best options. I take these reasons to be medically sound, although one can imagine cases of doctors steering patients toward medically bad decisions.

#### Recommending

I characterize *recommending* as the doctor providing her professional opinion about what the patient should choose. Recommending differs from informing, which means telling the patient about each option’s risks and benefits––in principle, as neutrally as possible––*without* the doctor offering her opinion. Thus, when recommending, the doctor makes a clear statement about which option she views as best, or the option about which a medical consensus has been reached.

Case A–recommending:

A patient with previous myomas, which were removed surgically, is pregnant for a second time. As contractions during labour increase the risk of life-threatening uterine rupture for patients with her condition, the doctor recommends a C-section, but leaves it up to the patient to decide: “It’s *advice,* but if you tell me that… ‘I’d really like to give birth’... […] then, of course, we will try to make it happen.”^5^ The patient––in collaboration with her partner, who is also present––opts for a C-section.

The doctor here gives explicit reasons (e.g., risk of uterine rupture) for recommending a C-section. This aligns with Oxman et al.’s definition of *recommending* as suggestions based on explicit reasons. However, I believe that this would be a case of recommending even if the doctor had not provided reasons why he believed a C-section is preferable.

A doctor merely *recommending* treatment leaves it up to the patient (and/or their family members if they are involved) whether to follow the recommendation or not. In Case A, the patient makes a decision according to the doctor’s recommendation. If the patient instead were to prefer a vaginal birth, the doctor would have had two options: either accept her choice or continue arguing for a C-section. If the latter course was taken, the conversation would have evolved from recommending to what I call (attempted) convincing and possibly (attempted) persuading, and would not count as simply recommending anymore.

The actual scenario (below, in bold) is compared to hypothetical scenarios of attempted convincing and persuading to illustrate the differences. (Table 1).

**Table 1:** Recommending compared to convincing and persuading

Recommending	Convincing	Persuading
**The doctor explains that a C-section is the medically preferred option due to lower risks. The patient chooses a C-section.**	The doctor explains that a C-section is the preferred option, but the patient wants a vaginal delivery. The doctor elaborates on the medical reasons for a C-section, emphasizing that it is the safest choice for her and the baby. In light of the reasons given, the patient reconsiders and concludes that she wants a C-section.	The doctor explains that a C-section is the preferred option, but the patient wants a vaginal delivery. The doctor elaborates on the medical reasons for a C-section, emphasizing that it is the safest choice for her and the baby. Despite the patient nonetheless wanting a vaginal delivery, she consents to a C-section.

#### Convincing

A patient who is convinced––after initially being reluctant about a treatment––has changed, rearranged, or clarified his treatment preferences in light of the reasons the doctor has given for treatment. Convincing is not merely about getting the patient to opt for a certain treatment course, but changing the patient’s mind about it, including correcting misguided beliefs, based on rational arguments. Thus, the way I use the term *convinced* is akin to what Faden and Beauchamp (who do not distinguish *persuade* from *convince*) characterize as being *persuaded*, namely, coming to believe something through reason. *Convince* comes from the Latin *convincere*, meaning “to overcome decisively” (Online Etymology Dictionary 2022). What has been overcome for a convinced patient, in my conception, is his initial disinclination to treatment.

Case B–convincing:

A patient with colorectal cancer is given a choice between two equally effective drug treatments: Fliri and Flox. The doctor says that it is ultimately up to the patient, but suggests Flox because Fliri may cause diarrhea, and the patient’s medical history puts her at particular risk. However, the patient prefers Fliri as Flox causes numbness in the arms and legs which––according to what a previous doctor has told her––can leave people incapacitated. Diarrhea, on the other hand, is something that she thinks she can handle. The doctor explains that there are ways to avoid severe and incapacitating numbness, such as starting treatment gradually. Moreover, she tells the patient, diarrhea can get very serious, and she is quite confident that Flox is a better option for her. The patient then consents to Flox, saying she feels this is best. She adds that the doctor has provided more in-depth information than the doctor she previously talked to.

Here, the doctor begins with recommending one option (Flox), but the patient prefers another (Fliri), so the doctor tries to convince her, successfully. Of course, we cannot know for certain whether the patient in fact changed her treatment preferences. However, her statements that she now feels that it is best to go with Flox and the fact that the doctor has provided in-depth information, arguably suggest that she has, indeed, changed her preferences in light of the doctor’s arguments.

It is worth noting that the patient’s preferences are not necessarily purely rational, as people’s preferences generally aren’t (Thaler and Sunstein 2009). In the present case, the patient’s initial preference for Fliri is based on her wanting to avoid becoming incapacitated, which may partly have an emotional component, such as fear. When the doctor convinces her to choose Flox by providing rational reasons in its favour, this may have affected how she feels about it by resolving her fear. Thus, the doctor’s *rational* arguments may have had an *emotional* effect on the patient.

Again, compare the actual scenario (in bold) to hypothetical scenarios of recommending and persuading to see the differences. (Table 2).

**Table 2:** Convincing compared to recommending and persuading

Recommending	Convincing	Persuading
The doctor recommends Flox, and the patient chooses Flox. / The doctor recommends Flox, but the patient wants Fliri. The doctor accepts Fliri as the chosen option.	**The doctor recommends Flox, but the patient wants Fliri. The doctor then provides more extensive reasons in favour of Flox. In light of these reasons, the patient changes her mind and consents to Flox.**	The doctor recommends Flox, but the patient wants Fliri. The doctor then provides more extensive reasons in favour of Flox, but the patient still wants Fliri. Nonetheless, she consents to Flox.

Distinguishing convincing from persuasion (or similar terms), although often missed in the literature on medical ethics as well as on paternalism more specifically, is far from new. The philosopher Hans Skjervheim refers to Socrates’ distinction between an opinion *with* insight versus an opinion *without* it when distinguishing convincing from persuading (Skjervheim 1996). According to Skjervheim, convincing––as opposed to persuading^6^––is about promoting insight, not about making the other person change their mind regardless of the reasons for doing so. Building on Skjervheim’s distinction, the Norwegian political scientist Einar Øverbye argues that being convinced of something means making the other’s reasons one’s own (Øverbye 2013). Applied to Case B, this would mean that the patient is convinced to choose Flox if she internalizes the doctor’s reasons for doing so. However, I believe this would raise the bar too high for characterizing something as convincing. Medicine is a complicated field, and patients will not always understand medical reasons well enough to internalize them. Coming around to preferring Flox because of the doctor’s argument that it does not cause severe diarrhea is, in my view, sufficient for being convinced, although the doctor may rely on a more complex set of medical reasons.

#### Persuasion

While the convinced patient has changed his mind about treatment, the persuaded patient has not. When a patient is persuaded––the way I use the term––he consents to treatment contrary to what he prefers (or what he, on some level, thinks he prefers). Thus, despite consenting to the treatment the doctor considers preferable, the patient remains reluctant. Rather than *changing* or *clarifying* preferences (which convincing is about), persuading involves the doctor *overriding* the patient’s preferences, for example, by dismissing them as irrelevant. Persuading, in my terminology, may involve the doctor providing rational arguments but not necessarily so. *Persuade* comes from the Latin *persuadere*, which means “to bring over by talking” (Online Etymology Dictionary 2022). One might interpret this as akin to what I have called convincing, for example, the patient being “brought over” to preferring a particular treatment, through arguments. The way I use the term *persuasion*, however, the patient is “brought over” to consenting––not by the arguments themselves but by the doctor’s efforts to make him do so. It follows from my descriptions that while a patient who is convinced is likely to have consented autonomously, a patient who is persuaded is not.^7^

Case C – persuasion:

A patient meets with a doctor to decide between a spinal block and narcosis for an upcoming kidney stone operation. A spinal block is preferable, the doctor says, but the patient wants narcosis because he dreads the spinal block needle, as well as being awake during surgery. The doctor is attentive to the patient’s fears and asks several questions to get to the bottom of them, yet also argues in favour of a spinal block: It is the most commonly used option, she says, the patient has likely had it in the past and doesn’t even remember it (implying that it can’t have been that bad), and he can get sedatives and not be awake, along with pain management before the needle insertion. The patient listens but continues to prefer narcosis. The doctor does a physical examination, then states: “Your lungs sound fine, so that’s good… […] So, I think it’s worth trying the spinal block.” The patient is hesitant and asks about the possibility for narcosis again, but the doctor argues that narcosis involves giving him so much medication that he would stop breathing, “which is actually scary in itself”. Finally, the patient consents with hesitation to a spinal block.

In this case, the doctor *recommends* a spinal block, then she tries to *convince* the patient, before finally *persuading* him. The patient continues to express a preference for narcosis despite the doctor repeatedly arguing for a spinal block. When the patient finally consents to the latter, he does not express doing so because he has changed his preferences in light of the arguments for the spinal block. To be sure, he may acknowledge the doctor’s arguments as good reasons to choose a spinal block, and he may not be completely opposed to the idea, but perhaps remains ambivalent between the medical reasons for a spinal block and a feeling that narcosis is more comfortable. However, he expresses a preference, first and foremost, for narcosis. Thus, there is reason to believe––or at least let’s say so for the sake of the argument––that the patient’s opting for a spinal block is mainly due to the doctor asking him to do so, rather than having changed his mind.

Once more, compare the actual scenario (in bold) to the hypothetical scenarios of recommending and convincing. (Table 3).

**Table 3:** Persuading compared to recommending and convincing

Recommending	Convincing	Persuading
The doctor says a spinal block is preferable, and the patient opts for a spinal block. / The doctor says a spinal block is preferable, but the patient wants narcosis. The doctor accepts narcosis as the chosen option.	The doctor says a spinal block is preferable, but the patient wants narcosis. The doctor provides reasons for a spinal block and the patient, due to the doctor’s arguments, consents to a spinal block.	**The doctor says a spinal block is preferable, but the patient wants narcosis. The doctor argues extensively for a spinal block. Despite the patient still wanting narcosis, he consents to a spinal block.**

In Case B, the doctor’s rational arguments may have exerted an emotional effect on the patient by resolving her fear. In Case C, by contrast, the patient’s fear of a spinal block––which is essential for why he prefers narcosis––remains. Nonetheless, the doctor’s persuasive efforts may exert an emotional effect on the patient. He might feel that it is easiest to forego his own preferences and give in, or he might want to please his doctor, which is not uncommon for patients (Hollander and Greene 2019; Klitzman 2007). In the literature on persuasion, the term *rational persuasion*––involving rational arguments––is sometimes used (e.g., Tsai 2014). I, however, speak only of *persuasion* as neither the doctor’s persuasive efforts nor the effect it has on the patient are necessarily (purely) rational.

#### The subtleties of different forms of influence

So far, I have sketched out what, for the sake of simplicity, I call three forms of influence: recommending, convincing, and persuading. In the next section, I will argue that persuading––as opposed to convincing and recommending––is paternalistic, but first, some aspects of these forms of influence need to be clarified.

The first point I want to make is that it is, indeed, artificial to sharply categorize and separate forms of influence as I have done above. Communication is an area of subtleties. Conversations flow back and forth––some parts may resemble convincing, others persuading. Also, in the previous section, I argued that being convinced involves overcoming doubts or disinclination regarding treatment, while being persuaded involves remaining reluctant. But what about patients who are ambivalent, which many––understandably––may be? Does being *convinced*, as I defined it, allow for ambivalence, or are patients who remain ambivalent when consenting *persuaded*? My suggested answer is to look at the degree of ambivalence. If a patient has serious doubts, he is more likely to have been persuaded. If he has *more or less* overcome doubts or disinclination, he is more likely to have been convinced. This admittedly is a vague differentiation, but the line between convincing and persuading is not clear-cut.

The second point I would like to address is how the issue of conflicting preferences may be resolved. In case B, exemplifying convincing, the patient’s preferences may have been *rearranged* (she went from preferring Fliri to preferring Flox) in light of the doctor’s arguments. Thus, the patient may have come to deem her preference for getting better without unnecessary risks (pointing to Flox) as more important than her preference for avoiding incapacity (pointing to Fliri). She may, however, have been somewhat ambivalent if both preferences remained. An alternative way of looking at it is that she––rather than rearranging her preferences––*disregarded* her initial preference (for Fliri) as she came to consider it ill-founded in the light of the arguments against it. If so, she may not have been ambivalent in her choice. As touched on above, rearranging or disregarding preferences may have an emotional, as well as a rational component, for example, by her fear of the consequences of the Flox treatment being resolved.

In Case C, on the contrary, the patient’s fear of a spinal block––and thereby his preference for narcosis––seems to remain. From the perspective of a hierarchical conception of preferences, the patient may have had a first-order (immediate) preference for being unconscious during the procedure, which points to narcosis, but a higher-order (overall) preference for avoiding risks, which points to a spinal block. How can his consenting to a spinal block be explained, then? The answer may lie in other preferences or inclinations––*not* related to the treatment course––such as wanting to avoid confrontation with the doctor. Thus, not only preferences related to treatment play a part in the decision-making process. Preferences or inclinations related to the doctor-patient relationship may also come into play.

In fact, something similar might have happened in case B as well. To add some meat to the bones of case B: When consenting to the Flox treatment, the patient said, “You are the one, of course, with the competence and experience, so I’ll do it.” This quote suggests that she may not have consented to Flox merely because of the doctor’s arguments after all, and that, rather, the patient’s trust in her doctor may have contributed. This does not necessarily mean she was persuaded in the sense of consenting against her preferences. Instead, she might have distanced herself from both her initial treatment preference *and* the doctor’s arguments and decided on laying her faith in the doctor. Thus interpreted, it may be objected that this case is not, in fact, an example of convincing after all, as I claimed above. However, the added quote only goes to show that––as argued above––the lines between convincing and persuading are in practice blurry.

My third point is that it may not make complete sense to place the different forms of influence at a fixed spot in the continuum ranging from non-controlling to controlling (or vice versa). As recognized by Faden and Beauchamp (1986), manipulation, for example, may be involved in parts of a conversation that are not generally characterized as manipulation. Adding meat also to the bones of case A, initially used as an example of recommending, illustrates this point. In reality, the doctor continued the conversation in a way that––through his emphasis on certain words––can be interpreted as manipulative nudges (although not necessarily intentional on the doctor’s part): “You may […] be at risk of an acute C-section [if not choosing a planned C-section], which is not our best advice, but if you *really* want to give birth [vaginally], you [wouldn’t be doing] anything *very* wrong in wanting it.” Moreover, due to the power asymmetry between doctor and patient, what may be meant merely as a recommendation by a doctor could be experienced as difficult to resist by patients. Thus, although *recommending*, in theory, may be placed close to the “non-controlling” end of the continuum, in practice, it can have a “controlling” effect. In fact, the “controlling” effect of recommending, where the doctor offers an opinion that is potentially difficult to resist, can be greater than that of convincing, for example when convincing mainly entails correcting the patient’s misguided beliefs. Also, different forms of influence work on different levels––some bypassing reason (e.g., manipulative nudges such as framing information to make a patient consent), while others do not (e.g., compulsory treatment). It is not obvious that manipulative influences––which the patient may be unaware of and therefore unable to object to––should be characterized as less controlling or autonomy-undermining than clear cases of coercion that the patient *is* aware of.^8^

Thus, neither sharp distinctions between forms of influence, nor generalizations about how controlling they are, make complete sense in practice. However, my aim is not to provide an accurate description of how treatment conversations work in practice, but rather to clarify the line between non-paternalistic and paternalistic communication and the latter’s ethical dimension. For this purpose, I believe that it can nonetheless be useful to operate with separate categories of influence placed on a continuum, although this is admittedly a less-than-perfect encapsulation.

### How can persuasion be paternalistic?

In the introduction, I loosely characterized paternalism as overriding another’s preferences with the intention of benefitting them or protecting them from harm. My characterization is a simplified version of Beauchamp and Childress’ definition of paternalism as “the intentional overriding of one person’s preferences or actions by another person, where the person who overrides justifies the action by appeal to the goal of benefiting or of preventing or mitigating harm to the person whose preferences or actions are overridden” (2019, 231–232). While many definitions include coercion or restriction of freedom or autonomy, with Dworkin’s (2020) as one of the most influential, Beauchamp and Childress’ definition and my slightly differing version do not explicitly do so. Thus, it may be objected that Beauchamp and Childress’ definition, as well as mine, fail to capture the essence of paternalistic action. However, overriding preferences may arguably be seen as undermining autonomy. Alternatively, one may argue from a motive-based account of paternalism like that of Shiffrin (2000) or Quong (2011). From a motive-based perspective, paternalism does not necessarily involve any restriction of freedom or autonomy. Instead, the paternalist’s motive––namely, that of viewing themselves as superior––is at the core of what paternalism is. One reason, according to Shiffrin, is that definitions including freedom or autonomy restriction as a necessary criterion render many actions that intuitively seem paternalistic, non-paternalistic. Dworkin makes a similar point by providing an example of a wife hiding her sleeping pills from her suicidal husband (2020). Although Dworkin, in contrast to motive-based accounts, includes restriction of autonomy or freedom in his characterization of paternalism, he takes this example as illustrating a paternalistic action, although not obviously involving restriction of freedom or autonomy.

A different objection to my suggested characterization of paternalism is that paternalism does not necessarily involve *overriding* preferences, but *disregarding* them or, as Groll (2012) formulates it, not taking the other’s will as decisive. For example, if one person does something to help a friend without consulting him, this is, in Groll’s view, paternalistic even if the friend, in fact, would have wanted help. In some contexts, this may be a reasonable view of paternalism. However, a definition emphasizing not taking the other’s will as decisive, rather than overriding their preferences, renders very many medical encounters instances of paternalism. Every time a patient arrives with an issue (say, a bacterial infection) and the doctor prescribes a treatment course (such as antibiotics) without asking the patient what he wants to do, the doctor, according to such a definition, is acting paternalistically. Rather than considering this action as paternalistic, I believe it should be seen as an inherent part of medical practice. Operating with a definition that renders common medical scenarios instances of paternalism, risks, in my view, trivializing the concept and the ethical dilemmas related to it.

For now, two main features of my characterization of paternalism should be highlighted. First, the paternalist goes against the other’s preferences or will, which in some sense or another is central to many much-cited accounts of paternalism (Groll 2012). Further, I follow Shiffrin (2000) and Dworkin (1988, 123) in emphasizing that by acting paternalistically, the paternalist substitutes her judgment for that of the person interfered with. In other words, the paternalistic doctor substitutes her patient’s judgment when overriding his preferences. When speaking of *preferences* in the following, I refer to *expressed* preferences unless otherwise specified. Second, the paternalist is guided by an intention to benefit the person whose preferences are overridden, which––although contested by Shiffrin (2000)––is also a common assumption about paternalism.

Certainly, my characterization of paternalism could be problematized further and there are many other definitions out there (see, e.g., Dworkin 2013)––each with their own strengths and limitations. However, I do not propose to attempt a definition that captures all instances of paternalism in general––or even all instances of paternalism in doctor-patient communication (lying for patients’ benefit, for example, is not necessarily captured by my characterization). And for the purpose of discussing doctors’ communication with patients reluctant about treatment, the above characterization will, in my view, do the job.

There is widespread agreement that recommending and convincing are not paternalistic, unless done in a manipulative way (for example, if the doctor deliberatively frames or withholds information to make the patient believe that a specific treatment course is the best one). Providing medical advice is at the core of the medical profession and part of what doctors are expected to do. As Savulescu formulates it, a doctor should not be merely “a fact-provider but also an argument-provider” (Savulescu 1995, 330). And a common view in paternalism literature is that rational persuasion––that is, making someone believe something through arguments––is not paternalistic; sometimes it is even explicitly contrasted to paternalism (Tsai 2014). Shiffrin illustrates the supposed contrast between rational persuasion and paternalism with the following example: B asks his acquaintance, A, for help building a shelf set, but A thinks he should practice doing it himself. If A were to refuse to help without explaining why, she would be acting paternalistically. If she explains instead why she does not want to help, she is not acting paternalistically, according to Shiffrin. Her argument is that by providing reasons, A shows respect for B’s reasoning capacities. Paternalism, by contrast, means taking over the other’s reasoning––substituting the other’s judgment and viewing one’s own as superior––which is what happens if A refuses to help without giving reasons (Shiffrin 2000). However, Shiffrin does not discuss how we should think about a situation where A provides reasons for not helping B, B replies that he does not find A’s reasons convincing and still wants her help, but A continues to refuse. This would resemble what happens in Case C, where the doctor provides reasons for a spinal block, the patient nonetheless continues to prefer narcosis, yet the doctor stands firm in what she views as the best option. Shiffrin is correct that the shelf-situation does not *begin* with a paternalistic action, and neither does Case C. However, in practice, conversations are dynamic and evolve. What may begin as a conversation with no clear paternalistic undertones could turn into an instance of paternalism over the course of a few sentences. Before I continue and explain how, let’s take a closer look at Tsai’s position.

Along with Shiffrin, Tsai emphasizes that the paternalist considers their own judgment superior. The paternalist thus perceives the person she interferes with as being less capable of realizing––or acting according to––what is best for him. Tsai goes against the grain and argues that rational persuasion can be paternalistic, or at least morally problematic in the same way that paternalism is. He defines rational persuasion as “(i) presenting another person with reasons, evidence, or arguments in favor of some attitude, belief, or action, (ii) performed in order to promote, or not undermine, rational decision-making or change of mind” (Tsai 2014, 90). It should be noted that Tsai’s analysis concerns interpersonal relationships such as friends or spouses and not the doctor-patient relationship, although he is open to his position being applicable to other contexts as well. He characterizes paternalism as “behavior aimed at promoting another person’s good that treats her like a child, or someone who cannot be trusted to look after her own good” (Tsai 2014, 78) and presents the following analysis of paternalistic rational persuasion:

[…] I treat you paternalistically in offering you reasons […] if, in doing so, I (i) am motivated by the distrust and concern, (ii) convey that you are insufficiently capable of canvassing or weighing reasons for yourself, and (iii) occlude an opportunity for you to independently engage with the reasons (2014, 80).

From Tsai’s perspective, the interpersonal paternalist thus considers his own judgment superior, acts from concern, distrusts the other person’s capacities to reason about different alternatives––and treats her accordingly––and prevents her from reasoning on her own. For the medical paternalist, on the other hand, I do not think that this description necessarily applies. If we are to perceive paternalistic doctors as “motivated by distrust” in their patients, we may be ascribing an unreasonably sceptical attitude to doctors. Further, if a doctor considers her own medical judgment to be superior to that of her patient, this is probably explained by the fact that the doctor usually *is* superior epistemically in medical matters due to years of medical education and practice. In other words, believing one’s judgment to be superior to the patient’s is hardly characteristic for paternalistic doctors in particular. Moreover, Tsai’s emphasis on independent reasoning does not obviously fit the medical paternalist––patients will often need help with reasoning on medical options without thereby being subject to paternalistic care. Thus, I believe there is a need for an analysis of paternalistic persuasion for the doctor-patient relationship specifically, which differs from the interpersonal paternalistic rational persuasion that Tsai discusses.

To sum up, a paternalistic doctor, on my account, overrides the patient’s preferences to benefit or protect him from harm, thereby substituting his judgment. Now, let’s return to Case C, where the doctor persuades the patient to opt for a spinal block despite his preferring narcosis. I view this as a case of paternalism. First, it can be assumed that the doctor’s goal is to protect the patient from harm as she argues against narcosis due to its risks. Second, I believe that this is a case of overriding preferences. The *Cambridge Academic Content Dictionary* defines *overriding* as “to ignore or *refuse to accept a suggestion*, idea, or method that already exists or operates” (emphasis added) (Cambridge Dictionary 2022). When the doctor in Case C asks the patient to consent to a spinal block––contrary to his preferences––this is not, first and foremost, an expression of her ignoring or not caring about his preferences, for which she indicates attentiveness and sympathy. What it is an expression of, is her not approving of narcosis as the option to choose. The patient has formed a preference and, thus, has an idea of how he would like the procedure to happen, but the doctor––at least in some sense––*refuses to accept* it. More precisely, she refuses to accept it as decisive for treatment. Third, when the doctor first provides reasons for choosing a spinal block in an attempt to change the patient’s mind (convince him), she involves his ability to make a judgment and does not act paternalistically. However, when her attempts at convincing him do not work, and he does *not* simply opt for a spinal block, she says *she* thinks that a spinal block is worth trying. The patient has formed a judgment of narcosis as the preferred option, but as this does not correspond to the doctor’s perception of the preferred option, she substitutes his judgment (that narcosis should be chosen) with her own (that a spinal block should be chosen).

In addition to Tsai, there are others who can be interpreted as supporting a view of persuasion as being paternalistic or compromising the freedom or interests of the person who is persuaded. Tsai quotes Dworkin as stating, “there are no methods of influencing people that are necessarily immune to being used paternalistically” (Tsai 2014, 111). Skjervheim does not discuss paternalism specifically, but implies that persuasion means going beyond the other’s freedom^9^ (Skjervheim 1996). And Oxman et al. argue that public health information that aims to persuade people risks compromising their ability to make informed choices (Oxman et al. 2022).

### The line between communicative paternalism and non-paternalism

In the previous section, three positions on the relationship between persuasion and paternalism were outlined. The common view holds that rational persuasion is not paternalistic as the person persuading appeals to the other’s reasoning abilities. Tsai’s position, on the contrary, is that rational persuasion can be paternalistic if the paternalist is guided by concern and distrust, conveys that the other is insufficiently capable of reasoning, and occludes their ability to reason independently. My position, set out above, holds that persuasion is generally paternalistic, while convincing is not. It should be emphasized, however, that *rational persuasion* commonly (see, e.g., Faden and Beauchamp 1986)––and also according to Tsai––is understood as providing reasons to make someone believe something. This is a somewhat different conception of *persuasion* than my conception of the term; what is often called *rational persuasion* lies closer to what I call *convincing*.^10^

Can rational persuasion, as it is commonly defined, be paternalistic––and is persuasion, as I have defined it, always so? I believe that the common position is correct that the act of rationally persuading someone, which resembles what I call convincing––when merely involving non-manipulatively providing arguments and appeals to the other’s reasoning––is not paternalistic. However, it follows from how I defined the three forms of influence (recommending, convincing, and persuading) that a doctor cannot know for certain whether she has persuaded or convinced a patient. Moreover, the doctor might try to persuade a patient but end up convincing him. Or, perhaps more likely, she might try to convince the patient, or merely mean to offer a recommendation, but end up persuading him, for example, because he regards the doctor as an authority he is reluctant to oppose. In the latter case, however, the doctor has not acted paternalistically in the sense of overriding––defined as *refusing to accept*––the patient’s preferences or substituting his judgment. Thus, although I have argued that persuasion is paternalistic, I believe there are exceptions––that persuasion is *not* paternalistic if the doctor simply meant to recommend, or convince (essentially, *rationally persuade*) a patient of, a treatment. Similarly, there are exceptions to convincing being non-paternalistic: If the doctor tries to persuade a patient she acts paternalistically even if he ends up convinced (essentially, *rationally persuaded*). Moreover, manipulatively convincing a patient can be paternalistic (on the common terminology, a scenario involving manipulation does not count as *rational persuasion*). If we stick with the common conception of rational persuasion, a doctor *rationally persuading* a patient, or trying to do so, is not paternalistic, while I believe the case of a patient *being rationally persuaded* can take place in the context of a paternalistic consultation. The latter can happen, for example, if the doctor dismisses and overrides (refuses to accept) the patient’s preferences, but the patient nonetheless is ultimately rationally persuaded by the reasons themselves.^11^

I term the form of paternalism occurring in communication *communicative paternalism.*^12^ A full account of what communicative paternalism entails would include several forms of paternalistic influences, such as manipulation and nudging, and perhaps also, for example, communicating health policies to the public. However, this is not my present goal. The point is to separate communicative paternalism––more specifically, persuasion––from communicative non-paternalism, such as recommending and convincing. The abovementioned exceptions aside, while persuasion is paternalistic in the sense of overriding preferences and substituting judgment, this generally does not happen, or so I have argued, during convincing or recommending. When a doctor merely recommends something, the patient’s preferences––which may or may not be affected by the doctor’s recommendation––will guide the decision. During attempted convincing, the patient’s initial preferences are not straightforwardly followed, yet they are also not overridden, as the goal is exactly that his (new) preferences––although changed or clarified since he entered the conversation––guide the decision. Thus, if relying on the previously presented continuum, the line between non-paternalism and paternalism generally falls between convincing and persuading.

What makes the doctor persuade a patient, and what makes the patient opt for treatment that he does not want? Part of the answer to the first question may be that the doctor believes that what the patient says he wants does not reflect what he really wants. In other words, she may believe that the patient has higher-order preferences that align with what the doctor views as medically sounder, which means that his first-order preferences would not be autonomous or authentic according to some hierarchical conceptions of autonomy (Sjöstrand and Juth 2014; Taylor 2005). Overriding these first-order preferences would be an act of soft paternalism. Alternatively, the doctor may think that although the patient truly wants something, and his first- and higher-order preferences and values align, he *should* want something else. Or she might not focus on the patient’s preferences at all, only on what she herself thinks best which would make her overriding his preferences––if, in fact, autonomous or authentic––a case of hard paternalism (Sjöstrand and Juth 2014).

The other part of the answer as to why doctors persuade patients, and why patients are persuaded, lies in the power asymmetry of the doctor-patient relationship. This asymmetry is widely discussed in the literature as it lies at the heart of many medical-ethical issues and has gained new relevance in, for example, the recent debate on epistemic injustice in healthcare (Carel and Kidd 2014). Both doctor and patient might perceive the doctor as epistemically superior due to her medical expertise (which she certainly is in medical matters), although not thereby necessarily having all the answers to what the individual patient needs. When patients give in and opt for treatment against their preferences, as we saw above, this may happen in a context in which the patient perceives the doctor as, in some sense, superior.

Is persuasion a form of pressure? Well, it depends on who you ask. A doctor might say that she merely encourages her patient to do what is best. She might even explicitly add that, when trying to persuade a patient, the decision is ultimately up to him. However, even if, strictly speaking, she does not insist on the treatment in question, the patient may perceive the doctor as insisting. Thus, the doctor’s persuasive efforts could be done in a way resembling––or be experienced by the patient as––what Savulescu describes as to “argue coercively” (Savulescu 1995, 330). This might create pressure that the patient finds difficult to resist, which admittedly can also be the case for patients experiencing attempts at convincing, recommending, or even informing, but more understandably so for patients being persuaded. Thus, there is potentially a kinship between persuasion and pressure and, therefore, it makes sense to place persuasion closer to the middle of the continuum, rather than at the non-controlling end.

Characterizing persuasion as coming close to pressure––potentially resulting in consent given non-autonomously––raises the question of whether consent given after persuasion is valid, as it requires voluntariness (Faden and Beauchamp 1986). However, a full discussion of this issue lies beyond the scope of this paper.

### What’s the problem with paternalistic persuasion?

Most theorists contend that paternalism is *prima facie* objectionable, although not always wrong. There are two main views on why paternalism is problematic (Begon 2016; Cornell 2015). The first is that paternalism is problematic because it undermines freedom or autonomy: The paternalist keeps the person whose autonomy is interfered with from acting according to his wishes. I will call this view *the autonomy view* of the problem with paternalism. The second view is held by motive-based theorists, who consider paternalism as not necessarily involving the restriction of autonomy or freedom. Instead, they argue, the problem of paternalism lies in the paternalist viewing himself as superior to the other: The paternalist treats the other as someone who lacks the capacities of autonomous agency, like a child unable to understand what is good for them, which is disrespectful or insulting (Begon 2016). I will refer to this view as *the motive-based view* on what is problematic about paternalism.

I believe that the autonomy view is the closest to capturing the problem with paternalistic persuasion, as well as many other instances of medical paternalism, and that the motive-based view misses the mark. Let’s take a clear (although probably quite unlikely) example of paternalism, inspired by an example by Groll (2012): a doctor operating on a patient against his will. The biggest problem here is arguably not the supposed insult (which seems like an understatement when facing coercive surgery) of being viewed or treated as less capable than the doctor to make medical decisions. The biggest problem seems to be being operated on without wanting to be.

Or let’s return to Case C and imagine that the kidney stone patient was not satisfied with the spinal block decision, yet, had a spinal block instead of narcosis. Although we obviously have no way of knowing how he felt, it seems reasonable that getting the spinal block would be worse than having his preference for narcosis dismissed, which may or may not have been experienced as insulting or disrespectful.

Instead, I believe that communicative paternalism is problematic for two reasons, both of which are partly captured by the autonomy view. The first problem occurs during the conversation and lies in the doctor dismissing the patient’s preferences as unimportant to the decision. Thus, what the patient experiences as important or valuable is not given a role in the decision-making process. The second problem is that if the doctor succeeds in persuading a patient, the patient may undergo treatment that he does not want (unless he changes his mind before the treatment is administered). I contend that in many cases, the latter problem is the biggest. In cases such as Case C, for example, it is problematic that the patient’s preferences are dismissed during the conversation with the doctor. But what is arguably more problematic is––to put it graphically––having a needle inserted into one’s spine without wanting it. It can be argued that the patient’s autonomy is undermined in two ways: first, during the treatment conversation and, second, when the treatment is effectuated. In accordance with the autonomy view, I believe that undermining patient autonomy is the main problem with paternalistic persuasion. However, I also believe that *undermining autonomy* can be understood in two ways and that in many cases, one of them is more problematic than the other.

This does not mean that I believe the motive-based view of the problem with paternalism is always wrong. But I do believe that the problem with paternalism is context-dependent and, as touched on by Coons and Weber (2013) and Schramme (2015), that different cases pose different ethical issues.

It follows from my discussion that “unsuccessful” attempts at persuading the patient are problematic, but less so than “successful” attempts. Further, I believe it is ethically relevant whether the doctor––if persuading a patient––means to do so or not. Certainly, the effect is the same in either case: The patient consents contrary to his preferences. However, just as a doctor has hardly acted objectionably even if a patient feels pressured by a mere recommendation, she generally has not, in my view, acted objectionably if she, when trying to convince or offering a recommendation to a patient, ends up persuading him. What is problematic is the doctor attempting persuasion, that is, dismissing and overriding the patient’s preferences.

That persuasion is problematic does not mean that doctors should never try to persuade patients. After all, the common view––with good reason––is that paternalism can be acceptable and that the patient’s expressed preferences should not be followed at all times. Faden and Beauchamp argue that it may be blameworthy *not* to try to persuade when medically required (Faden and Beauchamp 1986), and others (e.g., Conly 2013) have argued that *not* protecting people from themselves––rather than paternalism itself––is what is disrespectful. Some have proposed criteria for acceptable paternalism, such as the paternalistic act being likely to prevent the perceived harm, and the pros of paternalism outweighing the cons (see, e.g., Beauchamp and Childress 2019). Oxman et al. argue that persuasion aimed at making people change behaviour is more likely to be justified the more certainty there is about the option that the person is persuaded to choose, the greater the risk by choosing otherwise, and the greater the impact it has on others (Oxman et al. 2022). However, the problem with such criteria––aside from theoretical objections––is that it may not be realistic for doctors to remember and manage to act in accordance with them during conversations with patients. Perhaps this applies particularly to the difficult cases of patients being reluctant about treatment. If the doctor is focusing on remembering and applying ethical criteria, her attentiveness towards the patient might conceivably be compromised. I will therefore not suggest specific criteria for when––or how––persuasion is acceptable. What I will suggest is that paternalistic persuasion should not be undertaken lightly when patients express reluctance for particular treatments.

### Conclusion

The biggest problem with a doctor persuading a patient, which in my view is paternalistic, is that the patient may undergo treatment that he does not want, although it is also problematic that the doctor dismisses the patient’s preferences as unimportant. This does not mean that doctors should never try to persuade patients, but that it should not be undertaken lightly. Doctors should be critically aware of the fine line between non-paternalism and paternalism––a line that is crossed after shifting from recommending or trying to convince a patient to choose a treatment, to persuading him.

My discussion does not aim at demonstrating that other analyses and moral discussions about paternalism necessarily get it wrong, but rather that they do not sufficiently capture communicative paternalism––or more specifically, persuasion––in the doctor-patient relationship. This poses the question of whether it is, in fact, possible to develop a general analysis of paternalism suitable for all contexts, or general criteria for when it is acceptable. Paternalism theorists should acknowledge that there may not be a one-size-fits-all answer to the question of what is problematic with paternalism, which should be viewed as a more multi-faceted concept than some accounts allow for.

## Footnotes

1 For the sake of readability, I mostly refer to the doctor as she and the patient as he. However, gender has not been found to be significant in whether a doctor is paternalistic (Falkum and Førde 2001)

2 For an overview of accounts that take the view that persuasion is not paternalistic, see Tsai (2014).

3 In Faden and Beauchamp’s continuum, controlling influences are placed on the left end and non-controlling influences on the right. However, I follow Oxman et al. in placing non-controlling influences on the left end and controlling on the right, which is natural if perceiving the continuum as a stepwise process in time.

4 This number does not include cases where the patient merely hints at an opposing preference.

5 All quotes are translated from Norwegian. It should be noted that the patient did not initially express reluctance about a C-section.

6 The Norwegian word for what I call *convince* is *overtyda*/*overbevise*, and the word for what I call *persuade* is *overtala/overtale*.

7 However, this issue is not straight-forward. For example, let’s say a patient consents to treatment not because of the arguments but because he feels pressured to do so, yet the treatment aligns with what on hierarchical accounts of autonomy are considered higher-order preferences. Some might see this as an instance of persuasion that is consistent with autonomy. Moreover, a patient who is convinced as defined above might have changed his preferences due to manipulation, meaning he does not consent autonomously. However, the latter case is arguably not merely an instance of convincing but also of manipulation. Similarly, it may be argued that the former case is not a clear case of persuasion but, perhaps, lying somewhere between persuasion and convincing.

8 I would like to thank the anonymous reviewers for several of the specifications in this section.

9 More specifically, he claims that convincing––as opposed to persuading––does not mean going beyond the other’s freedom.

10 Tsai’s––as well as, for example, Faden and Beauchamp’s (1986)––conception of *rational persuasion* differs from my conception of *convincing* in that the former involves promoting rational decision-making––which excludes the possibility of manipulation––while the latter can involve manipulation, as explained in footnote 7.

11 Arguably, it follows from my discussion––with its emphasis on whether the doctor *means* to persuade or not––that my conception of paternalism implies that the paternalist *intentionally* overrides preferences, which would be in line with, for example, Beauchamp and Childress’ definition of paternalism. However, adding intentionality as a criterion raises the question of how conscious the doctor must be of his overriding for this criterion to be satisfied––an issue that requires a more thorough discussion than the present context allows for.

12 When using the term *communicative paternalism* in an earlier draft, I could not at the time find this concept used in any other sources. However, it was brought to my attention that Øverbye (2013) uses the same term in Norwegian (“kommunikativ paternalisme”) in approximately the same way, only in the context of benefit receivers in the welfare state, not in the doctor-patient relationship.
